# Supplementary material for: The interactome of intact mitochondria by cross-linking mass spectrometry provides evidence for coexisting respiratory supercomplexes
Source: Mol Cell Proteomics. 2017 Dec 8;17(2):216–32. doi: 10.1074/mcp.RA117.000470 (PMC5795388; doi:10.1074/mcp.RA117.000470)
Supplement: Supplemental Data [file supp_RA117.000470_133922_0_supp_23978_fzffwf.zip › spectra_annotation/mito_spectra_annotation/22-1-12-1-4-1.pdf]

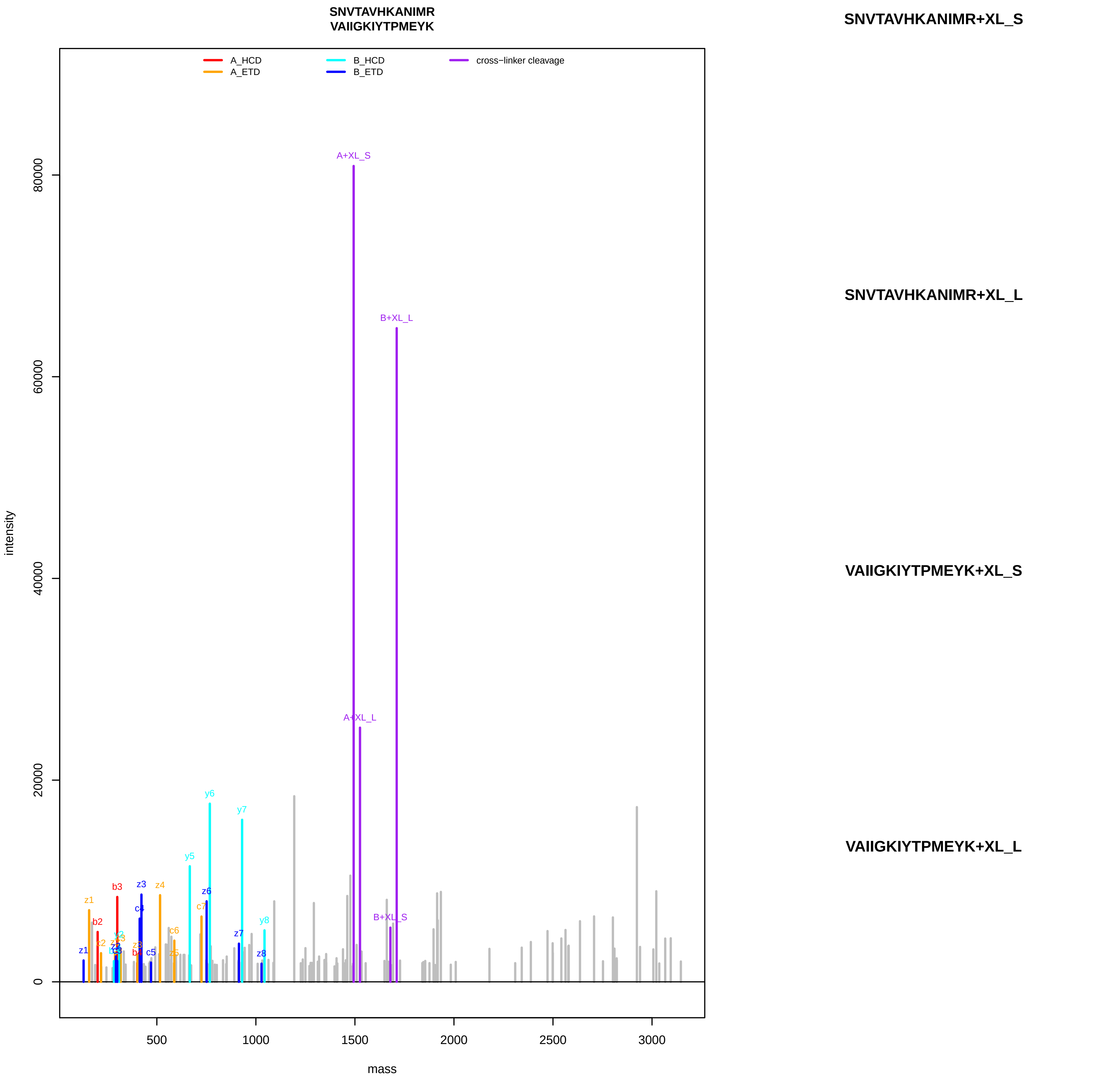
SNVTAVHKANIMR
VAIIGKIYTPMEYK
A_HCD
A_ETD
B_HCD
B_ETD
cross−linker cleavage
A+XL_S
B+XL_L
A+XL_L
B+XL_S
y6
y7
y5
y8
z1
b2
b3
z3
c4
z4
z6
c7
c6
z7
z8
c5
80000
60000
40000
20000
0
intensity
500
1000
1500
2000
2500
3000
mass
SNVTAVHKANIMR+XL_S
SNVTAVHKANIMR+XL_L
VAIIGKIYTPMEYK+XL_S
VAIIGKIYTPMEYK+XL_L
